# Neonatal Diabetes in Patients Affected by Liang-Wang Syndrome Carrying KCNMA1 Variant p.(Gly375Arg) Suggest a Potential Role of Ca^2+^ and Voltage-Activated K^+^ Channel Activity in Human Insulin Secretion

**DOI:** 10.3390/cimb43020073

**Published:** 2021-08-31

**Authors:** Chiara Mameli, Roberta Cazzola, Luigina Spaccini, Valeria Calcaterra, Maddalena Macedoni, Paola Azzurra La Verde, Enza D’Auria, Elvira Verduci, Gianluca Lista, Gian Vincenzo Zuccotti

**Affiliations:** 1 Department of Pediatrics, Vittore Buzzi Children’s Hospital, 20154 Milan, Italy; chiara.mameli@unimi.it (C.M.); valeria.calcaterra@asst-fbf-sacco.it (V.C.); maddalena.macedoni@asst-fbf-sacco.it (M.M.); enza.dauria@unimi.it (E.D.); elvira.verduci@unimi.it (E.V.); gianvincenzo.zuccotti@unimi.it (G.V.Z.); 2 Department of Biomedical and Clinical Sciences “L. Sacco”, Università di Milano, 20157 Milan, Italy; 3 Clinical Genetics Service, Vittore Buzzi Children’s Hospital, 20154 Milan, Italy; luigina.spaccini@asst-fbf-sacco.it; 4 Pediatric and Adolescent Unit, Department of Internal Medicine, University of Pavia, 27100 Pavia, Italy; 5 Division of Neonatology, Department of Pediatrics, Vittore Buzzi Children’s Hospital, 20154 Milan, Italy; paola.laverde@asst-fbf-sacco.it (P.A.L.V.); gianluca.lista@asst-fbf-sacco.it (G.L.); 6 Department of Health Sciences, Università di Milano, 20146 Milan, Italy

**Keywords:** big potassium channel, neonatal diabetes, insulin secretion, KCNMA1-linked channelopathy, Liang-Wang syndrome

## Abstract

Liang-Wang syndrome (LIWAS) is a polymalformative syndrome first described in 2019 caused by heterozygous mutation of the KCNMA1 gene encoding the Ca^2+^ and voltage-activated K^+^ channel (BKC). The KCNMA1 variant p.(Gly356Arg) abolishes the function of BKC and blocks the generation of K^+^ current. The phenotype of this variant includes developmental delay, and visceral and connective tissue malformations. So far, only three cases of LWAS have been described, one of which also had neonatal diabetes (ND). We present the case of a newborn affected by LIWAS carrying the p.(Gly375Arg) variant who manifested diabetes in the first week of life. The description of our case strongly increases the frequency of ND in LIWAS patients and suggests a role of BK inactivation in human insulin secretion. The knowledge on the role of BKC in insulin secretion is very poor. Analyzing the possible mechanisms that could explain the association of LIWAS with ND, we speculate that BK inactivation might impair insulin secretion through the alteration of ion-dependent membrane activities and mitochondrial functions in β-cells, as well as the impaired intra-islet vessel reactivity.

## 1. Introduction

The large-conductance Ca^2+^ and voltage-activated K^+^ channel (BKC) is a tetramer consisting of four α-subunits encoded by the KCNMA1 gene on chromosome 10q22.3 that in human co-assembles with modulatory β subunits that modify the channel functional properties and pharmacology [1]. BKCs are distributed in both excitable and non-excitable cells where they couple the increase in intracellular Ca^2+^ concentration ([Ca^2+^]i) to hyperpolarization of the membrane potential playing key roles in the control of cellular excitability and in the maintenance of K^+^ homeostasis and cell volume in non-excitable cells [2]. They exert a pleiotropic action in many physiological processes, such as the control of neuronal excitability, neurotransmitter and hormone release, vascular tone, innate immunity [3,4,5,6] and pathological states related to cancer [7,8,9]. BKC are localized both in the plasma membrane and intracellular membranes; their activation allows the transit of large amounts of K^+^ across the membranes and plays a negative feedback controller role on the activities of other membrane channels, including the voltage-dependent Ca^2+^ channels VDCC [10]. BKC openers stabilize the cell by greatly increasing K^+^ outflow and leading to hyperpolarization of membranes, and, as a result, decrease cellular excitability and/or cause smooth muscle relaxation.

The dysfunctions of BKCs can generate alterations in different tissues and organs, among which the best-described concern the smooth muscle, the kidney, the nervous system, and the cochlea [1]. The electrical activity mediated by the passage of ions through the cell membranes is fundamental for the release of insulin from β-cells, but K^+^ regulates this process mainly through the activity of the ATP-sensitive K^+^ (K_ATP_) channel [11]. K_ATP_ channels are metabolic sensors that can couple the cell’s metabolic status with electrical activity to regulate many cellular functions by controlling the cell membrane potential [11]. In pancreatic β-cells, K_ATP_ channels modulate insulin secretion in response to fluctuations in plasma glucose levels, and thus are important regulators of glucose homeostasis. Even if BKCs were electrophysiologically identified in primary and clonal β-cells more than 30 years ago and in human β-cells in 2008 by Braun et al. [6], there is poor knowledge on BKC activity and insulin secretion.

Liang-Wang syndrome (LIWAS, OMIM **#**618729) is a polymalformative syndrome first described in 2019 determined by loss-of-function variants of the KCNMA1 gene, which encodes the BKC α-subunits. Of those, the KCNMA1 variant p.(Gly356Arg) was found to abolish the function of the BKC and to block the generation of K^+^ current [3]. A phenotypic analysis of three patients (one boy and two girls aged 3, 21 and 12 years, respectively) carrying the p.(Gly375Arg) variant of the KCNMA1 gene revealed a novel syndromic neurodevelopmental disorder named LIWAS which was associated with severe developmental delay, visceral and cardiac malformations, and connective tissue abnormalities with arterial involvement, bone dysplasia and characteristic dysmorphic features [3]. Neonatal diabetes (ND) occurred in only 1 of the 3 LIWAS patients [3]. Given the low prevalence of ND, the association between ND and LIWAS was considered casual, and ND was not included in the clinical features of this new syndrome. To the best of our knowledge, no other new cases of LIWAS have been reported since; therefore, the association between these two diseases has yet to be confirmed. We present a new case of a newborn affected by LIWAS carrying the p.(Gly375Arg) variant who manifested diabetes in the first week of life. Our case strongly increases the frequency of ND in LIWAS patients suggesting a role of BKC inactivation in human ND.

## 2. Case Presentation

Herein, we report the case of a newborn affected by LIWAS carrying the KCNMA1 (p.(Gly375Arg) variant. The newborn was a female born at 35 + 5 weeks of gestation from non-consanguineous parents. Neonatal anthropometry was normal (birth weight 2685 g, length 48 cm), as was her Apgar score (a quick test performed on a baby at 1 and 5 min after birth that determines how well the baby tolerated the birthing process). The pregnancy was characterized by the detection of polyhydramnios, an overdistended stomach, bilateral hydronephrosis, dilation of intestinal loops, clitoral hypertrophy and bilateral microphthalmia at 31 weeks of gestation. At birth, the newborn presented several congenital abnormalities in addition to those detected in the prenatal period: microcephaly, hypertelorism, complete intestinal malrotation and prepyloric antral membrane, unique urogenital sinus, and a large patent ductus arteriosus with significant systemic to pulmonary shunt, leading to pulmonary hypertension. The neurological picture was characterized by hyperexcitability, hypotonia and a poor repertoire of spontaneous movements with normal brain magnetic resonance imaging. At 3 days of life, she developed persistent insulin-dependent hyperglycemia (>250 mg/dL), leading to a diagnosis of neonatal diabetes (ND). Abdominal ultrasound revealed normal pancreas morphology. After 72 h of life, total parenteral nutrition was started and continued until she died. She was treated with insulin (insulin requirement 0.01–0.05 U/kg/h) and she wore a continuous glucose-monitoring device. Her metabolic control was good (HbA1c: 51 mmol/mol, 6.8%). She also developed respiratory failure requiring invasive mechanical ventilation and she underwent three surgical interventions to correct the heart and the intestinal malformations. Following the last abdominal surgery, she developed fatal sepsis due to Serratia, which led to exitus at 3 months of age. A few days prior to death, the genetic analysis of the KCNMA1 gene showed the heterozygous variant c.1123G > A. The protein variant determines the p.Gly375arg amino acid change which defines the Liang-Wang syndrome.

## 3. Discussion

To the best of our knowledge, ND was reported only in one child diagnosed aged 3 years carrying the KCNMA1 p.(Gly375Arg) variant, whereas the other two patients diagnosed at a later age (12 and 21 years) were not affected by ND [3]. However, the report of Liang and colleagues did not give any clinical information and records about diabetes history [3]. The description of our case strongly increases the frequency of ND in patients with LIWAS and suggests that ND could be one of the clinical characteristics of this syndrome, as well as that the lack of ND in other patients could be ascribed to an incomplete penetrance of genotype into phenotype as in other genetic diseases [12]. We therefore propose including endocrine evaluation in newborns with a strong clinical suspicion of LIWAS in the first days of life to make the diagnosis of ND at an earlier stage.

It is known that the dysfunction of other K^+^ channels, such as the K_ATP_ channel [13,14] and proton-sensitive TALK 1 channel [15,16], leads to ND. Undoubtedly, our clinical observation prompts the reconsideration of BKC’s role in the human pancreatic β-cells. The BKCs are unique amongst ion channels because of their dual activation by depolarizing membrane potentials and increased [Ca^2+^]i. They have a complex regulation pattern that includes the modulatory β-subunits, subunit phosphorylation (by protein kinases A, C, G and CaMKII), pH, and endogenous messengers (NO, cAMP, cGMP) and modulators (arachidonic acid, lipoxygenase and cytochrome P450 epoxygenase metabolites) [17]. Despite numerous experimental evidence suggesting that BKC channels play a fundamental and specific role in many conditions and represent a promising pharmacological target, several years of intense research effort in both academia and industry have not yet led to the approval of BKC modulators for clinical use [17]. 

Regarding diabetes, several studies have reported a diminished BKC-mediated vasodilation in coronary micro-vascularization of diabetic patients [18,19,20,21], probably resulting from harmful effects of blood glucose fluctuations leading to overproduction of reactive oxygen species (ROS) and alteration of the protein kinase C α/nuclear factor -κB/muscle ring fingers protein 1 signaling pathway [22] and the consequent ubiquitin-mediated degradation of the channel β1 subunit by the ubiquitin-proteasome system [18]. Unfortunately, the studies on the relationships between BKC inactivation and insulin secretion are very few and mainly performed in cellular and animal models by using channel blockers with different specificity, such as peptide toxins, the alkaloid paxilline and tetraethylammonium [23]. However, the different types of islet cells have distinct ion channel profiles and there are differences between human β cells and those of other mammals that must be taken into account [10]. Initially, these channels were thought to play a role in the metabolic enhancement of insulin secretion [24], but the contradictory results of subsequent investigations [25,26,27] and the lack of specific BKC reversible openers and blockers [6] have led to little and confusing knowledge in this topic. For this reason, we can only make assumptions about how the loss of activity of these channels could lead to ND.

To perceive the nutritional status, the β cells are grouped into islets that form a dense network with small blood vessels. Human islets represent 1–2% of the weight of the pancreas, have a complex architecture in which β-cells are directly juxtaposed to non-β-cells, but receive up to 20% of the pancreatic blood supply [10]. Islets have little innervation by cholinergic, adrenergic, and peptidergic nerve branches that, unlike in mice, in humans create contact with smooth muscle vascular cells (SVCs) rather than with endocrine cells [28]. Insulin secretion is regulated by endocrine, biochemical, and neuronal signals. At the endocrinal level, various hormones, such as melatonin, estrogens, leptin, growth hormone, glucagon-like peptide-1, and gastric inhibitory polypeptides regulate insulin secretion [29].

**Figure 1:**
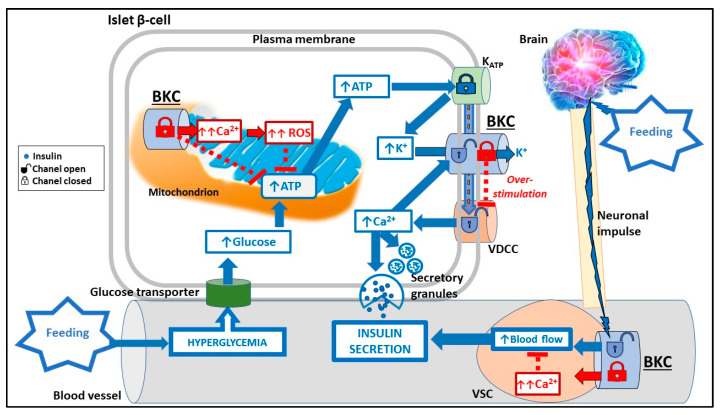
Simplified schematic representation of the effects of BKC inactivation in pancreatic β-cells. The inhibitory role of BKC inactivation (depicted in red) on some physiological mechanisms of insulin secretion (depicted in blue) evoked by hyperglycemia and autonomic impulses. Please see text for further details.

At the biochemical level, glucose metabolism stimulates insulin secretion by generating, triggering, and amplifying signals in β-cells. Insulin is secreted mainly in response to increased cytosolic ATP concentration derived from a fast and intense glucose uptake and catabolism [29]. β-cells share many characteristics with nerve cells including electrical excitability and changes in membrane potential couple changes in glycemia to insulin secretion promoted by Ca^2+^ influx through voltage-dependent Ca^2+^ channels (VDCCs). High glycolytic ATP synthesis and mitochondrial ATP export rapidly increase the ATP/ADP cytosolic ratio, triggering the closure of the K_ATP_ channel in the plasma membrane (Figure 1). The resulting plasma membrane depolarization increases the open probability of VDCCs, triggering an increase in cytosolic Ca^2+^ concentration that promotes exocytosis of insulin containing secretory granules (Figure 1). In the postprandial state, glucose metabolism generates additional signals which increase the amount of insulin secreted in response to the triggering Ca^2+^ influx [30]. The pentose phosphate pathway and the export of mitochondrial tricarboxylic acid cycle intermediates, such as isocitrate, increase the cytosolic NADPH and reduced glutathione which create signals that amplify insulin secretion [31]. The mobilization of Ca^2+^ from the endoplasmic reticulum (ER) also participates in the amplification of Ca^2+^ signaling. ER Ca^2+^ homeostasis is determined by ion movements across the ER membrane, including K^+^. Numerous K^+^ channels, including BKCs, have been detected in the membranes of the ER [32]. However, ER is the synthesis site of membrane proteins that are then transported to the Golgi apparatus and from the Golgi apparatus to their destination on the cell surface or elsewhere. The K^+^ permeable trimeric intracellular cation channels, which are found only in the ER [33], and potentially the ryanodine receptor [32], inositol 1,4,5-trisphosphate receptor [32], and TALK-1 channel [15] may contribute to the maintenance of the balance of charge movement that occurs during the release of Ca^2+^ from the ER, while the functionality of other K^+^ channels in the ER has not yet been clarified.

Lastly, at the neuronal level, the autonomic regulation of insulin secretion take place mainly indirectly via the stimulation of SVCs and the consequent increase in blood flow [34] (Figure 1). 

BKC activity can indirectly influence biochemical and neuronal regulation of insulin secretion. In various cell types, BKCs are part of the membrane repolarizing mechanism that helps reset the ionic balance to facilitate the generation of further action potentials and act as a kind of “emergency brake” [4]. Even if BKCs do not seem to affect bulk [Ca^2+^]_I_ in β-cells, the loss of their plasma membrane polarizing activity might leave more VDCC in the inactivated state, thereby affecting insulin exocytosis [26] (Figure 1).

On the other hand, BCKs might prevent Ca^2+^ overload in the mitochondria of β-cells and in SVCs of islet vessels. A study performed on BKC knockout mice demonstrated that the deletion of the KCNMA1 gene determines mitochondrial Ca^2+^ overload when β-cells were exposed to high glucose concentration. This increase in mitochondrial Ca^2+^ concentration determined a decrease in ATP synthesis and an increase in the levels of ROS and cellular apoptotic rate, leading to a reduction in insulin secretion and impaired glucose homeostasis in vivo [26] (Figure 1). In addition, SVCs under conditions supporting vasoconstriction, L-type Ca^2+^ channel (LTCC) activity in sarcoplasmic reticulum increases [Ca^2+^]_I_, and consequently promotes cell contraction, while, under conditions favoring vasodilation, the BKC activation by hyperpolarizing the membrane leads to an inhibition of LTCC [35]. In the human pancreas, BKC activation by neuronal impulses favors the dilation of contracted intra-islet vessels, thereby increasing blood flow and promoting insulin release [10,18,36] (Figure 1). 

Bearing in mind the above considerations, it is reasonable to assume that the loss of BKC function caused by the KCNMA1 gene mutation may have indirectly impaired insulin secretion by altering the regulation of membrane electrical activity and consequently decreasing the number of activated VDCC, the ATP-dependent closure of K_ATP_ channels, the β-cell proliferation and/or the islet perfusion in our patient. Since the response to glucose matures during the first postnatal days [37], this alteration of pancreatic functions at this time point may have led to overt diabetes. 

Finally, the establishment of the KCNMA1-channelopathy consortium has truly pushed BKC activators and blockers as a therapeutic target for several neuronal and other possible diseases [36]. The development of these drugs could open new therapeutic perspective for patients affected by LIWAS. However, no data are currently available on this syndrome, especially for use in the pediatric population. More studies are needed to expand the knowledge on their use in patients affected by this rare disease. The strength of our work is the unique perspective from which we started to analyze the possible relationships between BKC activities and insulin secretion in humans, while the main limitation is the lack of experimental evidence. However, the present study paves the way for a reconsideration of the role of BKCs in human insulin secretion, as well as for the development of new BKC activators and blockers which could have positive therapeutic effects on KCNMA1-linked channelopathies and other disorders associated with alterations of BKC activity.

## 4. Conclusions

Our report shows that a multitude of complex and inter-related pathways determined by BKC inactivation (alteration in functions of β-cells and SVCs) could impair insulin secretion. We suggest considering the KCNMA1 p.(Gly375Arg) variant as a mutation potentially associated with ND in LIWAS and propose including baseline endocrine evaluation at the time of diagnosis and in the ongoing monitoring of patients affected by this rare syndrome. New studies focused on the role of BKCs in human insulin secretion are warranted.

## Data Availability

Not applicable.
